# Enriched Environment Suppresses Neuronal Ferroptosis Through SIRT1/AKT/GSK3β-Dependent Glycogen Metabolic Reprogramming After Cerebral Ischemia–Reperfusion

**DOI:** 10.3390/antiox15050570

**Published:** 2026-04-30

**Authors:** Bao Zhou, Yixi Hao, Pengkun Yang, Haocheng Qin, Zheng Zhang, Na Ren, Lu Sun, Zhengran Ding, Zhong He, Shuai Zhang, Zijian Hua, Ya Zheng, Ce Li, Shenyi Kuang, Yulian Zhu, Kewei Yu

**Affiliations:** 1 Department of Rehabilitation Medicine, Huashan Hospital, Fudan University, Shanghai 200040, China; 23211220070@m.fudan.edu.cn (B.Z.); 25211220068@m.fudan.edu.cn (Y.H.);; 2 National Center for Neurological Disorders, Shanghai 200040, China; 3 Department of Neurology, Huashan Hospital, Fudan University, Shanghai 200040, China

**Keywords:** cerebral ischemia-reperfusion injury, glycogen metabolic reprogramming, oxidative stress, ferroptosis, enriched environment

## Abstract

Neuronal ferroptosis is a key contributor to secondary brain injury following cerebral ischemia, yet the metabolic mechanisms governing this process remain poorly understood. Enriched environment (EE) is a housing paradigm that provides enhanced sensory, cognitive, and social stimulation through complex physical surroundings and increased opportunities for voluntary activity. Our preliminary data indicate that EE confers cerebroprotection against ischemia-induced ferroptosis; however, whether this effect is associated with glycogen metabolic regulation and the underlying molecular pathways has not been elucidated. This study aimed to determine whether EE may influence ferroptosis-associated pathways, potentially via Sirtuin 1 (SIRT1)/protein kinase B (AKT)/glycogen synthase kinase-3β (GSK3β)-related mechanisms of glycogen metabolism. Using a mouse model of middle cerebral artery occlusion (MCAO) and an oxygen–glucose deprivation/reoxygenation (OGD/R) cellular model, we performed behavioral assessments, molecular and biochemical analyses, and pharmacological interventions to elucidate mechanistic pathways. EE was associated with improved neurological outcomes and reduced infarct volume after ischemia. Mechanistically, EE appeared to activate the SIRT1/AKT pathway and increase the inhibitory phosphorylation of GSK3β and relieving its suppressive effect on glycogen synthase, which may underlie the observed increase in glycogen levels within ischemic brain tissue. Pharmacological inhibition of SIRT1 largely diminished these metabolic and neuroprotective benefits. Consistently, at the cellular level, SIRT1 overexpression contributed to the restoration of glycogen metabolism and robustly attenuated ferroptosis under ischemic conditions. Collectively, these findings suggest that EE may attenuate ferroptosis-related pathways possibly involving SIRT1/AKT/GSK3β-dependent glycogen metabolic remodeling, providing a novel metabolic perspective on EE-induced cerebroprotection and highlighting SIRT1-centered regulation of glycogen metabolism as a potential therapeutic target for ischemic stroke.

## 1. Introduction

Stroke is a major acute cerebrovascular disorder, with ischemic stroke accounting for approximately 87% of all cases, and remains a substantial global health burden due to the resulting neuronal loss and long-term neurological impairments [1]. Current clinical management primarily relies on vascular recanalization strategies, including intravenous thrombolysis and mechanical thrombectomy [2]. However, despite their efficacy in restoring blood flow, reperfusion often precipitates secondary cerebral ischemia–reperfusion injury (CIRI), a process in which oxidative stress plays a central pathological role [3]. During reperfusion, the abrupt overproduction of reactive oxygen species (ROS) triggers lipid peroxidation, protein oxidation, and DNA damage, collectively accelerating neuronal death [4]. Thus, oxidative stress-driven cell death has become a critical determinant of neurological outcome following ischemic stroke.

Ferroptosis, a recently characterized form of iron-dependent regulated cell death distinguished by overwhelming lipid peroxidation, has emerged as a key mechanism contributing to neuronal loss after CIRI [5]. When antioxidant defenses, particularly the activity of glutathione peroxidase 4 (GPX4), are compromised, the cellular capacity to detoxify lipid peroxides diminishes, thus initiating ferroptotic death cascades. These findings highlight ferroptosis as a highly promising therapeutic target for neuroprotection in ischemic stroke [6]. However, the cellular and metabolic contexts that govern neuronal susceptibility to ferroptosis under ischemic conditions remain incompletely understood.

Maintenance of neuronal energy homeostasis is essential for resistance to metabolic stress. Glycogen, an important glucose storage form in the brain, has gained increasing attention for its role in cerebroprotection and metabolic resilience [7]. Traditionally, brain glycogen was believed to be confined to astrocytes; however, recent evidence suggests that neurons possess functional glycogen metabolism that dynamically adapts to ischemic stress [8]. Notably, ischemia/reperfusion-induced ferroptosis is characterized by excessive lipid peroxidation, and emerging evidence suggests [9] that metabolic state may influence susceptibility to this form of cell death. These findings suggest that glycogen metabolism may represent a critical metabolic hub linking cellular energy status to oxidative stress resistance. Nevertheless, its specific role in ferroptosis regulation and the upstream mechanisms controlling glycogen metabolic remodeling remain largely undefined.

The enriched environment (EE), originally conceptualized by Donald Hebb, is a well-established non-pharmacological intervention paradigm that involves enhanced sensory, cognitive, and social stimulation through exposure to complex housing conditions, including novel objects, increased physical activity, and social interaction [10,11]. Accumulating evidence indicates that EE exerts robust neurobiological effects in both physiological and pathological contexts. EE has been shown to promote synaptic plasticity and inhibit various forms of cell death, thereby facilitating neurological recovery following cerebral ischemia. Our previous work also indicated that EE enhances autophagic flux and improves mitochondrial quality control after stroke [12]. These effects suggest that EE exerts broad regulatory actions on cellular stress responses and metabolic homeostasis. Notably, recent studies suggest that metabolic reprogramming plays a critical role in determining neuronal vulnerability to ischemic injury. In particular, alterations in glucose utilization and glycogen metabolism may influence redox balance and lipid peroxidation processes, which are central to ferroptotic cell death [13]. However, whether EE can modulate central energy metabolic patterns—particularly glycogen metabolism—to influence ferroptosis has not yet been elucidated.

Sirtuin 1 (SIRT1), an NAD^+^-dependent deacetylase, serves as a central regulator linking cellular energy metabolism with stress responses [14]. SIRT1 has been reported to activate the protein kinase B (AKT) signaling pathway, leading to inhibitory phosphorylation of glycogen synthase kinase 3β (GSK3β) [15]. Given that GSK3β is a key negative regulator of glycogen synthase, its inactivation directly relieves the suppression of glycogen synthesis. Importantly, this signaling axis positions SIRT1 as a potential molecular bridge connecting EE, metabolic regulation, and cell fate determination.

Prior studies have shown that EE improves neurological recovery after cerebral ischemia/reperfusion and can attenuate neuronal ferroptosis, and enrichment-related signaling has been linked to the AKT/GSK3β axis. Nevertheless, whether these effects converge on glycogen metabolic remodeling, and whether SIRT1 acts as an upstream regulatory node in this process, remain unresolved. Based on these insights, we hypothesized that EE is associated with the SIRT1/AKT/GSK3β signaling axis and drives neuronal glycogen metabolic reprogramming (favoring glycogen synthesis and suppressing glycogen breakdown), thereby attenuating ferroptosis after ischemia/reperfusion. The present study aimed to systematically validate this hypothesis. Through combined in vivo and in vitro experiments, we provide evidence that EE modulates glycogen metabolism and attenuates ferroptosis-related processes through the SIRT1/AKT/GSK3β pathway. Furthermore, causal relationships were confirmed using SIRT1 inhibitors and other targeted interventions. These findings not only reveal a previously unrecognized metabolic mechanism underlying the neuroprotective effects of EE but also provide theoretical support and potential therapeutic targets for ischemic stroke treatment.

## 2. Materials and Methods

### 2.1. Experimental Animals

Sexually mature male C57BL/6 mice (8–12 weeks old, 24–28 g) were purchased from Zhejiang Vital River Laboratory Animal Technology Co., Ltd (Jiaxing, China). All experimental procedures strictly followed the National Institutes of Health (NIH) Guide for the Care and Use of Laboratory Animals. The study protocol was approved by the Experimental Animal Ethics Committee of Fudan University (Ethics Approval No. 2020-Huashan Hospital-JS-163). Mice were housed under standard laboratory conditions (22 ± 2 °C, 50 ± 10% humidity, 12 h light/dark cycle) with free access to food and water.

The sample size was estimated based on previous studies [16] using the MCAO model and our preliminary experiments, while aiming to minimize animal use in accordance with the 3Rs principle. Considering the complexity of the experimental design, multiple independent outcome measures, and an anticipated mortality rate of approximately 10–15% following MCAO surgery, a total of 270 mice were included to ensure sufficient statistical power across all analyses. Of these, 65 mice were allocated to the sham group, and the remaining animals were subjected to MCAO surgery. Due to the destructive nature of several experimental procedures, independent cohorts of animals were used for different experimental endpoints, and the sample sizes reported for each experiment correspond to the number of animals used specifically for that analysis. In addition, to ensure animal welfare, perioperative analgesia was provided according to institutional animal care guidelines.

Perioperative analgesia was administered using buprenorphine (0.05 mg/kg, subcutaneous injection) [17] immediately after surgery and every 12 h for 48 h thereafter, in accordance with institutional animal care guidelines. Animals were euthanized at predefined experimental endpoints, with all tissue collection performed after continuous intervention for 7 days. For euthanasia, mice were deeply anesthetized with isoflurane (5% for induction and maintained at 2–3% until loss of pedal reflex), followed by transcardial perfusion with cold saline. Death was confirmed by cessation of heartbeat and respiration prior to tissue collection.

### 2.2. Middle Cerebral Artery Occlusion (MCAO) Model

Mice were anesthetized with isoflurane (5% induction, 2% maintenance; RWD Life Science, Shenzhen, China). A midline cervical incision was made to expose the left common, external, and internal carotid arteries. After ligation of the external carotid artery, a silicone-coated nylon filament (diameter 0.20 mm; Guangzhou Jialing Technology Co., Ltd., Guangzhou, China) was inserted through the external carotid stump into the internal carotid artery to occlude the origin of the middle cerebral artery. Occlusion lasted for 60 min. Body temperature was maintained at 37.0 ± 0.5°C using a thermostatically controlled heating pad (Harvard Apparatus, Holliston, MA, USA). Regional cerebral blood flow (rCBF) was monitored with a laser Doppler flowmeter (PeriFlux System 5000, Perimed, Järfälla, Sweden). A reduction in rCBF of more than 80% relative to the contralateral side indicated successful occlusion [18]. After 60 min, the filament was withdrawn to achieve reperfusion. After surgery, animals were randomly assigned to experimental groups using a computer-generated randomization schedule. Investigators performing behavioral testing, histological analysis, and data quantification were blinded to group allocation throughout the experiments to minimize potential bias.

### 2.3. Antibody and Pharmaceutical Administration

In vivo SIRT1 inhibition was achieved using the selective SIRT1 inhibitor EX-527 [19,20] (MedChemExpress, Monmouth Junction, NJ, USA). Mice were treated with EX-527 at a dose of 10 mg/kg, administered intraperitoneally once daily for 7 days starting 24 h after MCAO surgery. The dose and treatment regimen were based on prior studies demonstrating the efficacy of EX-527 in inhibiting SIRT1 in ischemia–reperfusion models [21]. Mice in groups not receiving the inhibitor were administered an equivalent volume of the corresponding vehicle under the same schedule. In vitro, MZ-101 (MedChemExpress, Monmouth Junction, NJ, USA), a selective inhibitor of glycogen synthase 1 (GYS1), was used to inhibit glycogen synthesis in SH-SY5Y cells, thereby enabling assessment of the functional role of glycogen metabolic remodeling. Cells were treated with MZ-101 at 5 μM for 24 h before the oxygen-glucose deprivation/reperfusion (OGD/R) protocol. The concentration and treatment duration were optimized based on the manufacturer’s instructions and previous studies demonstrating its efficacy in inhibiting glycogen synthesis and modulating glycogen metabolic pathways [22]. Cells in the non-inhibitor groups were treated with the same concentration of the corresponding solvent as a vehicle control. Primary antibodies used are detailed in Supplementary Table S1.

### 2.4. Housing Conditions and Enriched Environment (EE)

After MCAO model induction, mice were randomly allocated into three groups: the standard housing intervention group (MCAO), the enriched environment intervention group (MCAO + EE), and the enriched environment combined with inhibitor treatment group (MCAO + EE + SIRT1^−^). EE exposure began on the second day after neurological assessment. The enriched environment provided various gustatory, visual, and tactile stimuli to enhance sensory, motor, and cognitive activity, including diverse bedding materials, spices (pepper, star anise, cinnamon, etc.), and novel objects such as running wheels, tunnels, building blocks, and shelters. Cages for EE (80 × 60 × 40 cm) housed approximately 12 mice, offering enhanced social interaction and activity space. The arrangement of objects was changed daily to maintain novelty. Control groups were housed in standard cages (27 × 22.5 × 18 cm) with 4–6 mice per cage [23].

### 2.5. Cell Culture and Oxygen–Glucose Deprivation/Reperfusion (OGD/R) Model

The SH-SY5Y human neuroblastoma cell line (CCTCC, Wuhan, China) was cultured in a 1:1 mixture of MEM (Gibco 11-140-050, Gibco, Grand Island, NY, USA) and Ham’s F12 (Gibco 21-700-075, Gibco, USA) supplemented with 10% fetal bovine serum (Gibco 10-100-147, Gibco, USA) and 1% penicillin–streptomycin (Thermo Fisher Scientific, Waltham, MA, USA). Cells were maintained at 37 °C in a humidified 5% CO_2_ atmosphere. For the OGD/R model, cells were incubated in glucose- and serum-free Hank’s balanced salt solution (HBSS; Cat#14025-092, Gibco, USA) inside a hypoxia chamber (MIC-101, Billups-Rothenberg, Del Mar, CA, USA) flushed with 95% N_2_ and 5% CO_2_ to achieve <0.2% O_2_. After 4 h of hypoxia, cultures were returned to normoxic conditions and complete medium for 24 h of reoxygenation [24].

### 2.6. Construction of SIRT1-Overexpressing SH-SY5Y Cells

Stable SH-SY5Y cell lines with constitutive overexpression of human SIRT1 were generated using a lentiviral transduction system. The full-length SIRT1 coding sequence was cloned into a lentiviral expression vector containing a puromycin resistance gene, and recombinant lentiviruses were packaged in HEK293T cells by co-transfection with the helper plasmids psPAX2 and pMD2.G using Lipofectamine 3000 (Thermo Fisher Scientific, USA). Culture supernatants containing viral particles were harvested at 48 and 72 h after transfection, clarified by centrifugation, and passed through a 0.45 μm membrane filter (Millipore, Billerica, MA, USA). For infection, SH-SY5Y cells were seeded in 6-well plates and exposed to the viral suspension supplemented with 8 μg/mL polybrene (Sigma-Aldrich, St. Louis, MO, USA). After 48 h, transduced cells were subjected to selection with puromycin (2 μg/mL) for 7–10 days to generate stable cell populations. Successful overexpression of SIRT1 was verified by quantitative real-time PCR and immunoblot analysis before subsequent oxygen–glucose deprivation/reoxygenation (OGD/R) experiments.

### 2.7. Assessment of Infarction Volume

Cerebral infarction volume was assessed using 2,3,5-triphenyltetrazolium chloride (TTC) staining (Sigma-Aldrich, St. Louis, MO, USA). Mice were deeply anesthetized and decapitated, and brains were quickly removed and sliced into 2 mm coronal sections. Sections were incubated in 2% TTC solution at 37 °C for 30 min in the dark, then fixed in 4% paraformaldehyde (Sigma-Aldrich, St. Louis, MO, USA). Infarct and normal areas were quantified with ImageJ software [25] (v1.53, NIH, Bethesda, MD, USA). Infarction volume was calculated as: Relative infarct ratio (%) = [(volume of healthy hemisphere − volume of non-infarcted area on ischemic side)/volume of healthy hemisphere] × 100.

### 2.8. Nissl Staining

Nissl staining was performed to evaluate neuronal morphology and survival. Paraffin-embedded brain sections were dewaxed, rehydrated, and stained with toluidine blue or cresyl violet solution (Solarbio, Beijing, China). Sections were differentiated in 95% ethanol and observed under a SlideView VS200 slide scanning system (Olympus Corporation, Tokyo, Japan). The density of Nissl-positive neurons was quantified in peri-infarct areas using ImageJ [26].

### 2.9. TUNEL Staining

Neuronal cell death in the peri-infarct region was evaluated using a TUNEL fluorescence staining kit (Thermo Fisher Scientific, Waltham, MA, USA) according to the manufacturer’s protocol. Briefly, brain sections were deparaffinized, rehydrated, and subjected to antigen retrieval, followed by incubation with the TUNEL reaction mixture containing terminal deoxynucleotidyl transferase (TdT) and fluorescein-labeled dUTP. To specifically identify neurons, TUNEL staining was combined with NeuN immunofluorescence labeling. Nuclei were counterstained with DAPI. Images of the peri-infarct area were acquired using a FluoView FV3000 confocal microscope (Olympus Corporation, Tokyo, Japan), and TUNEL-positive neurons were quantified in a blinded manner.

### 2.10. Immunofluorescence

Immunofluorescence staining was conducted on both tissue sections and cultured cells. For tissue samples, mice were perfused with saline and fixed in 4% paraformaldehyde one week after intervention. Brains were dehydrated, paraffin-embedded, and sectioned (4 μm). Sections were dewaxed, rehydrated, and subjected to antigen retrieval in citrate buffer (pH 6.0). After blocking with 3% goat serum for 1 h, sections were incubated with primary antibodies overnight at 4 °C, followed by fluorophore-conjugated secondary antibodies for 1 h in the dark. Nuclei were counterstained with DAPI. Fluorescence images were obtained using an FV3000 confocal microscope (Olympus, Tokyo, Japan). Cell immunostaining followed a similar procedure after fixation with 4% paraformaldehyde and methanol permeabilization. Fluorescence images were captured using identical microscope settings for all groups. Quantification of fluorescence intensity was performed using ImageJ software (NIH, Bethesda, MD, USA). Based on the mouse brain atlas (Paxinos and Franklin) and standardized anatomical landmarks, including the bregma level and cortical structures, regions of interest (ROIs) were consistently defined within the peri-infarct cortex. The peri-infarct region was identified as the cortical area immediately surrounding the infarct core, as determined by corresponding TTC staining or histopathological features. To ensure consistency, ROIs were selected from comparable coronal sections across all animals. At least three sections per animal and three fields per section were analyzed.

### 2.11. Western Blot Analysis

Protein expression in mouse peri-infarct cortical tissues and SH-SY5Y cells was examined by Western blotting [27]. For total protein extraction, samples were lysed in ice-cold RIPA buffer supplemented with protease and phosphatase inhibitors (BestBio, Shanghai, China) and homogenized on ice, followed by centrifugation at 12,000× *g* for 15 min at 4 °C to remove insoluble debris. The supernatants were collected as total protein extracts. Nuclear proteins were isolated using a Nuclear/Cytosol Fractionation Kit (BioVision, Milpitas, CA, USA) in accordance with the manufacturer’s instructions. Protein concentrations were determined using a BCA Protein Assay Kit (Beyotime, Shanghai, China). Equal amounts of protein (20–40 μg) were separated by 10–12% SDS–PAGE and transferred onto PVDF membranes (0.45 μm; Millipore, Billerica, MA, USA). After blocking with 5% non-fat milk in TBST for 1 h at room temperature, membranes were incubated overnight at 4 °C with primary antibodies (1:1000), followed by incubation with HRP-conjugated secondary antibodies (1:10,000; Yeasen, Shanghai, China) for 1 h at room temperature. Immunoreactive bands were detected using an enhanced chemiluminescence (ECL) kit (Yeasen, Shanghai, China) and quantified with ImageJ software (NIH, Bethesda, MD, USA).

### 2.12. Transmission Electron Microscopy

Peri-infarct cortical tissues (~1–3 mm^3^) were collected to examine the ultrastructure of mitochondria within neurons. Samples were immediately fixed in 2.5% glutaraldehyde in 0.1 M phosphate buffer (pH 7.4) at 4 °C for at least 3 h, followed by post-fixation in 1% osmium tetroxide (Electron Microscopy Sciences, Hatfield, PA, USA). Tissues were then dehydrated through a graded ethanol series and embedded in Spurr resin (Ted Pella, Redding, CA, USA). Ultrathin sections (~70 nm) were prepared using an ultramicrotome (Leica, Wetzlar, Germany), stained with 2% uranyl acetate (Polysciences, Warrington, PA, USA) and Reynolds’ lead citrate, and examined under a Talos 120C transmission electron microscope (Thermo Fisher Scientific, Waltham, MA, USA) operating at 120 kV. Images were captured from neurons within the peri-infarct region to assess mitochondrial morphology.

### 2.13. Mitochondrial Membrane Potential Assay

Mitochondrial membrane potential was assessed using JC-1 dye (T3168, Invitrogen, Carlsbad, CA, USA). SH-SY5Y cells were incubated with JC-1 (10 μg/mL) at 37 °C for 15 min in the dark, washed with PBS, and imaged using an Olympus CKX53 fluorescence microscope (Olympus Corporation, Tokyo, Japan). The red/green fluorescence ratio was calculated to evaluate mitochondrial depolarization.

### 2.14. Intracellular Ferrous Iron (Fe^2+^) Detection

Intracellular ferrous iron levels were assessed using a Cellular Red Fluorescent Ferrous Ion Assay Kit containing RhoNox-6 probe [28] (C1190M, Beyotime, Shanghai, China). RhoNox-6 is a selective fluorescent sensor that reacts specifically with Fe^2+^ in living cells, resulting in the generation of a red fluorescent signal proportional to intracellular Fe^2+^ concentration. SH-SY5Y neuronal cells were seeded into 6-well plates and cultured under standard conditions. After the designated treatments, cells were incubated with RhoNox-6 working solution for 30 min at 37 °C in the dark. Subsequently, cells were gently washed with PBS to remove excess probe, and fluorescence images were captured using a fluorescence microscope.

### 2.15. Reactive Oxygen Species (ROS) Detection

Intracellular ROS levels were measured using the DCFH-DA (2′,7′-dichlorofluorescein diacetate) probe (Beyotime, Shanghai, China). Cells were incubated with 10 μM DCFH-DA at 37 °C for 30 min, washed, and observed with a fluorescence microscope (excitation/emission 488/525 nm). ROS levels were quantified by mean fluorescence intensity.

### 2.16. Lipid Peroxidation Assays for MDA and 4-HNE

To evaluate lipid peroxidation in the peri-infarct cortex, malondialdehyde (MDA) and 4-hydroxy-2-nonenal (4-HNE) levels were quantified in tissue homogenates. Briefly, peri-infarct cortical samples were excised, weighed, and homogenized in ice-cold phosphate-buffered saline (PBS, pH 7.4). Homogenates were clarified by centrifugation at 12,000× *g* for 10 min at 4 °C, and the resulting supernatants were collected for analysis. MDA concentrations were determined using a thiobarbituric acid (TBA) lipid peroxidation assay kit (Nanjing Jiancheng Bioengineering Institute, Nanjing, China) by reacting MDA with TBA under heat to form a colored adduct, which was measured spectrophotometrically at 532 nm. 4-HNE content was measured with a competitive enzyme-linked immunosorbent assay specific for 4-HNE (Cell Biolabs, Inc., San Diego, CA, USA) following the manufacturer’s protocol. All assays were normalized to tissue weight and expressed as relative changes in MDA and 4-HNE levels across groups.

### 2.17. Quantification of UDP-Glucose (UDPG) in Peri-Infarct Region by LC-MS

UDPG levels in the peri-infarct region of mouse brain were determined by liquid chromatography–mass spectrometry (LC-MS) [29]. At the designated time points after ischemia, mice were euthanized and the peri-infarct cortical tissues were rapidly dissected on ice based on anatomical landmarks referenced to the mouse brain atlas (Paxinos and Franklin), snap-frozen in liquid nitrogen, and stored at −80 °C until analysis. Approximately 20–50 mg of frozen tissue was homogenized in ice-cold methanol/water (80:20, *v*/*v*) containing an internal standard using a tissue homogenizer (TissueLyser II, Qiagen, Hilden, Germany), followed by protein precipitation at −20 °C and centrifugation at 14,000× *g* for 15 min at 4 °C (Eppendorf 5430R, Eppendorf, Hamburg, Germany). The supernatants were collected, dried in a vacuum concentrator (Concentrator Plus, Eppendorf, Germany), and reconstituted in the initial mobile phase for LC-MS analysis. Chromatographic separation was performed on an ultra-high-performance liquid chromatography (UHPLC) system (Vanquish, Thermo Fisher Scientific, USA) equipped with a HILIC column (BEH Amide, 2.1 × 100 mm, 1.7 µm; Waters, Milford, MA, USA) using a water–acetonitrile gradient. Mass spectrometric detection was carried out on a high-resolution mass spectrometer (Q Exactive Orbitrap, Thermo Fisher Scientific, USA) with electrospray ionization operated in negative ion mode. Quantification was achieved using external calibration curves generated from UDPG standards (Sigma-Aldrich, USA) and normalized to tissue weight.

### 2.18. Glycogen Quantification

Cellular glycogen content was measured fluorometrically using the Glycogen Colorimetric/Fluorometric Assay Kit (Abcam, Cambridge, UK, #ab65620) according to manufacturer’s instructions [30,31].

### 2.19. Body Weight Monitoring

Body weight was recorded daily before surgery and throughout the post-operative and intervention periods using an electronic balance (Sartorius, Göttingen, Germany; accuracy ± 0.01 g). Changes in body weight were used to monitor overall metabolic and physiological status.

### 2.20. Neurological Function Assessment (mNSS)

Neurological function was evaluated using the modified Neurological Severity Score (mNSS) at 12 h post-operation and one week after intervention. The scale (0–18 points) assesses motor, sensory, reflex, and balance functions, with higher scores indicating more severe deficits [32]. Evaluations were performed by two blinded observers.

### 2.21. Neurobehavioral Assessment

After one week of the assigned interventions, behavioral evaluations were conducted on day 8 to examine functional recovery in each group. Motor performance was quantitatively characterized using three complementary paradigms, including gait analysis, open field assessment, and rotarod testing, providing an integrated evaluation of locomotor ability, spontaneous activity, and motor coordination. Evaluations were performed by two blinded observers.

#### 2.21.1. CatWalk XT Gait Analysis

Gait parameters were analyzed one week after intervention using the CatWalk XT system (Noldus, Wageningen, The Netherlands). Mice walked freely across a 150 cm illuminated glass walkway. Paw contact data were captured at 100 Hz, and three valid runs per animal were analyzed. Parameters included stance time, swing speed, stride length, base of support, and paw print intensity.

#### 2.21.2. Open Field Test

Mice were placed individually in a 40 × 40 × 60 cm open field apparatus. After 5 min adaptation, spontaneous activity was recorded for 10 min using SMART 3.0 software (Panlab, Barcelona, Spain). Parameters such as total distance, mean speed, and time spent in the center zone were analyzed to assess locomotor and exploratory behaviors [33].

#### 2.21.3. Rotarod Test

Mice underwent adaptive training for three days before surgery. One week after intervention, the fall latency on an accelerating rotarod (4–40 rpm over 5 min) was recorded, with 200 s as the inclusion criterion. Passive clinging was considered test termination.

### 2.22. Quantitative Real-Time PCR (qRT-PCR) Analysis

Total RNA was isolated from mouse cortical tissues or cultured neurons using TRIzol reagent (Invitrogen, Carlsbad, CA, USA) according to the manufacturer’s instructions. RNA concentration and purity were assessed with a NanoDrop 2000 spectrophotometer (Thermo Fisher Scientific, Wilmington, DE, USA). For cDNA synthesis, 1 μg of total RNA was reverse-transcribed using a commercial reverse transcription kit (Vazyme, Nanjing, China). Quantitative PCR was performed with ChamQ SYBR qPCR Master Mix (Vazyme, Nanjing, China) on a QuantStudio 6 Flex Real-Time PCR System (Applied Biosystems, Foster City, CA, USA). The thermal cycling program consisted of an initial denaturation at 95 °C for 30 s, followed by 40 cycles of 95 °C for 10 s and 60 °C for 30 s. A melting curve analysis was conducted at the end of amplification to verify primer specificity. Relative gene expression levels were calculated using the 2^−ΔΔCt^ method, with Gapdh serving as the internal control. The primer sequences (OriGene, Rockville, MD, USA) used in this study (5′→3′) were as follows:

Acsl4-F: CCTTTGGCTCATGTGCTGGAAC;

Acsl4-R: GCCATAAGTGTGGGTTTCAGTAC;

Gpx4-F: CCTCTGCTGCAAGAGCCTCCC;

Gpx4-R: CTTATCCAGGCAGACCATGTGC;

Gapdh-F: CATCACTGCCACCCAGAAGACTG;

Gapdh-R: ATGCCAGTGAGCTTCCCGTTCAG.

### 2.23. Determination of NADP^+^/NADPH

Intracellular NADP^+^ and NADPH levels were measured using a WST-8-based assay kit (Beyotime, Shanghai, China) following the manufacturer’s instructions [34]. Briefly, treated SH-SY5Y cells (~1 × 10^6^) were lysed in extraction buffer and centrifuged (12,000× *g*, 4 °C, 10 min). The supernatant was collected for analysis. For total NADP (NADP total), samples were directly assayed; for NADPH, aliquots were heated at 60 °C for 30 min to decompose NADP^+^. Samples (50 μL) were incubated with G6PDH working solution at 37 °C for 10 min, followed by color development using WST-8 reagent. Absorbance was recorded at 450 nm using a Multiskan™ FC microplate reader (1410101; Thermo Fisher Scientific, USA). NADP total and NADPH levels were calculated from a standard curve, and NADP^+^ content was obtained by subtraction.

### 2.24. Mitochondrial Viability Assessment

Mitochondrial viability was evaluated using Mito-Tracker staining as previously described [35], with minor modifications. Briefly, SH-SY5Y cells cultured on coverslips were rinsed with PBS after treatment and incubated with Mito-Tracker Red CMXRos (Beyotime, Shanghai, China) diluted to 50 nM at 37°C for 30 min in the dark. After staining, cells were washed and either directly counterstained with DAPI or subjected to fixation and subsequent immunofluorescence procedures. Fluorescence signals were captured using a fluorescence microscope or a confocal imaging system (Olympus FV3000, Olympus, Tokyo, Japan), and mitochondrial staining intensity was quantified accordingly.

### 2.25. Statistical Analysis

Continuous variables were first assessed for normality using the Shapiro–Wilk test and are presented as the mean ± standard deviation (SD) when normally distributed. Differences in body weight across time points and groups, as well as mNSS scores, were evaluated using two-way analysis of variance (ANOVA) followed by Tukey’s post hoc test for multiple comparisons. For all other datasets involving comparisons among multiple groups, one-way ANOVA with Tukey’s post hoc test was applied. For in vitro experiments, n represents independent experimental repetitions performed on separately cultured cells, rather than biological replicates from distinct donors. Each experiment was repeated at least three times independently to ensure reproducibility. For in vivo experiments, “n” represents the number of animals per group. Each animal was considered one independent biological replicate. For biochemical and molecular analyses requiring tissue homogenates, peri-infarct cortical samples obtained from the same animal were pooled to generate a single sample for analysis. Therefore, pooled tissues from one animal were treated as one biological replicate, and do not represent independent samples. For behavioral assessments, each animal was tested three times with sufficient rest intervals between trials, and the mean value was used as a single data point. For TUNEL staining, immunofluorescence, and cellular fluorescence analyses, three randomly selected fields of view per sample were imaged and averaged to represent one sample. For biochemical and molecular assays, including MDA and 4-HNE measurements, sample sizes were determined and increased based on established literature to ensure adequate biological replication.

All statistical analyses were conducted using SPSS Statistics version 22.0 (IBM Corp., Armonk, NY, USA) and GraphPad Prism version 8.0 (GraphPad Software, La Jolla, CA, USA). *p*-value < 0.05 was considered statistically significant, denoted as follows: ns, not significant; * *p* < 0.05; ** *p* < 0.01; *** *p* < 0.001; **** *p* < 0.0001.

## 3. Results

### 3.1. Enriched Environment Alleviates Neuronal Ferroptosis and Promotes Functional Recovery Following Cerebral Ischemia–Reperfusion Injury

**Figure 1:**
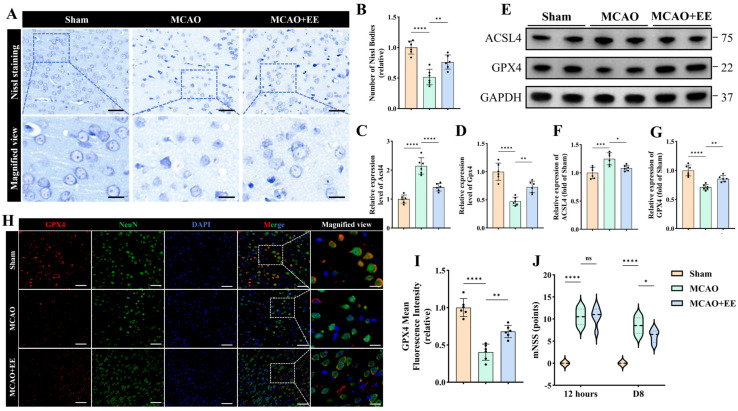
EE attenuates neuronal ferroptosis and may improve neurological outcomes after cerebral ischemia–reperfusion injury. (**A**) Representative Nissl staining images showing neuronal morphology and survival in the peri-ischemic cortex of Sham, MCAO, and MCAO + EE groups; scale bar: 50 µm (main images); 16.7 µm (magnified views). (**B**) Quantification of Nissl-positive neurons (*n* = 6). (**C**,**D**) Quantitative real-time PCR analysis of Acsl4 and Gpx4 mRNA expression in peri-infarct cortical tissue (*n* = 6). (**E**) Representative Western blot bands of ferroptosis-related proteins ACSL4 and GPX4. (**F**,**G**) Quantitative analysis of ACSL4 and GPX4 protein expression determined by Western blotting (*n* = 6). (**H**) Representative immunofluorescence images of GPX4 co-labeled with NeuN, illustrating GPX4 expression in cortical neurons; scale bar: 50 µm (main images); 16.7 µm (magnified views). (**I**) Quantification of GPX4 fluorescence intensity (*n* = 6). (**J**) Violin plots showing mNSS scores at 12 h and day 8 following ischemia (*n* = 6). All data are presented as mean ± standard deviation (SD). mNSS scores were analyzed using two-way ANOVA followed by Tukey’s post hoc test; all other group comparisons were analyzed using one-way ANOVA followed by Tukey’s post hoc test. The dashed boxes indicate the regions shown in the magnified views. ns, not significant; * *p* < 0.05, ** *p* < 0.01, *** *p* < 0.001, **** *p* < 0.0001.

To evaluate the neuroprotective effects of EE on cerebral ischemia–reperfusion injury (CIRI), we first examined neuronal morphology using Nissl staining. A mouse model of focal cerebral ischemia was induced by middle cerebral artery occlusion (MCAO) using the Longa intraluminal filament method. Compared with the Sham group, ischemic mice exhibited severe neuronal structural disruption in the peri-infarct region, characterized by shrunken cell bodies, blurred cellular outlines, and a substantial loss of Nissl bodies (Figure 1A). In contrast, EE markedly preserved neuronal morphology and structural integrity. Quantitative analysis further confirmed that the number of Nissl-positive neurons was significantly higher in the MCAO + EE group than in the MCAO group (*p* < 0.01, Figure 1B).

To further determine whether EE modulates ferroptosis-related molecular events after CIRI, key ferroptosis markers were subsequently assessed. Western blot analysis revealed a pronounced increase in the ferroptosis-promoting protein ACSL4 and a concomitant reduction in the ferroptosis-suppressing protein GPX4 following MCAO. Notably, EE intervention significantly reversed these pathological alterations, as evidenced by reduced ACSL4 levels and restored GPX4 expression (*p* < 0.05, Figure 1E–G). Consistently, quantitative real-time PCR analysis of peri-infarct cortical tissue demonstrated that Acsl4 mRNA expression was significantly elevated, whereas Gpx4 mRNA expression was markedly decreased after MCAO; both changes were markedly attenuated by EE exposure (*p* < 0.01, Figure 1C,D).

To validate these findings at the cellular level, immunofluorescence staining of GPX4 was performed (Figure 1H). Compared with the Sham group, GPX4 fluorescence intensity in MCAO mice was markedly diminished in neurons (*p* < 0.0001, Figure 1I), whereas EE markedly enhanced GPX4 expression in neuronal populations within the peri-infarct region (*p* < 0.01, Figure 1H,I).

Given that molecular and histological improvements do not necessarily translate into functional outcomes, neurological deficits were further evaluated using the modified neurological severity score (mNSS) [36]. MCAO markedly impaired neurological function at both 12 h and day 8 after ischemia (*p* < 0.0001, Figure 1J). At 12 h post-ischemia, no significant difference in mNSS scores was observed between the MCAO and MCAO + EE groups, indicating comparable baseline neurological deficits (*p* > 0.05, Figure 1J). In contrast, mice housed under EE conditions exhibited significantly improved neurological performance on day 8 compared with the MCAO group (*p* < 0.05, Figure 1J).

Collectively, these morphological, molecular, and behavioral data provide convergent evidence that EE effectively mitigates neuronal ferroptosis and promotes functional recovery following CIRI.

### 3.2. EE Promotes Adaptive Remodeling of Neuronal Glycogen Metabolism After Cerebral Ischemia–Reperfusion Injury

**Figure 2:**
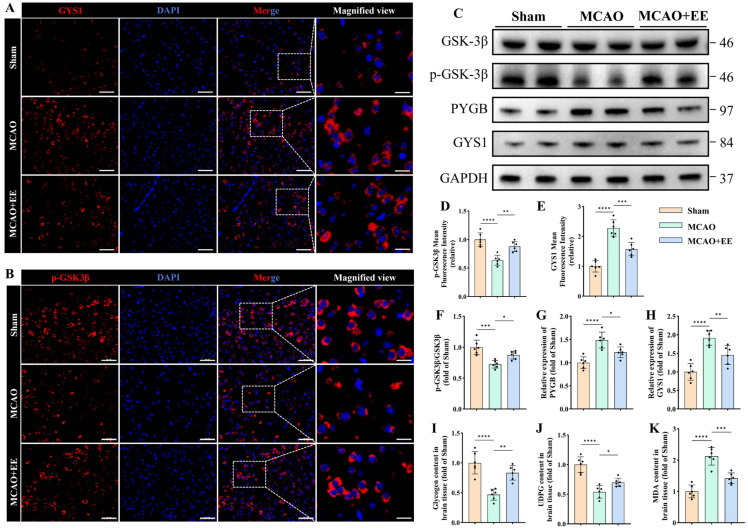
EE may improve glycogen metabolism in neurons following cerebral ischemia–reperfusion injury. (**A**) Representative immunofluorescence images of p-GSK3β; scale bar: 50 µm (main images); 16.7 µm (magnified views). (**B**) Representative immunofluorescence images of GYS1; scale bar: 50 µm (main images); 16.7 µm (magnified views). (**C**) Representative Western blot bands of GSK3β, p-GSK3β, PYGB, and GYS1. (**D**) Quantification of p-GSK3β fluorescence intensity (*n* = 6). (**E**) Quantification of GYS1 fluorescence intensity (*n* = 6). (**F**–**H**) Quantification of the p-GSK3β/GSK3β ratio, PYGB protein expression, and GYS1 protein expression (*n* = 6). (**I**) Quantitative analysis of glycogen content in the peri-infarct region tissue of mice (*n* = 6). (**J**) LC-MS quantification of UDPG levels in brain tissue (*n* = 6). (**K**) Quantification of MDA levels in brain tissue (*n* = 6). All data are shown as mean ± standard deviation (SD). Statistical comparisons were performed using one-way ANOVA followed by Tukey’s post hoc test. The white dashed boxes indicate the regions shown in the magnified views. * *p* < 0.05, ** *p* < 0.01, *** *p* < 0.001, **** *p* < 0.0001.

Accumulating evidence suggests that metabolic remodeling is closely associated with neurological recovery after cerebral ischemia–reperfusion, and glycogen metabolism has emerged as a critical component of neuronal metabolic homeostasis under ischemic stress [37]. To determine whether EE modulates glycogen metabolic signaling in the post-ischemic brain, the phosphorylation status of the upstream regulatory kinase GSK3β was first examined. Immunofluorescence analysis showed a marked reduction in p-GSK3β immunoreactivity in cortical neurons of the MCAO group compared with the Sham group, whereas EE exposure significantly enhanced p-GSK3β fluorescence intensity (*p* < 0.01, Figure 2B,D). Western blotting further validated this finding: the p-GSK3β/GSK3β ratio was significantly decreased following MCAO but markedly restored by EE intervention (*p* < 0.05, Figure 2C,F). These results indicate that ischemia suppresses the inhibitory phosphorylation of GSK3β, whereas EE reactivates this inhibitory modification, thereby favoring glycogen synthesis.

The expression of key enzymes involved in glycogen metabolism was next assessed. The glycogen-degrading enzyme PYGB was significantly upregulated in the MCAO group, while EE intervention markedly reduced its expression (*p* < 0.05, Figure 2G). In contrast, the protein level of phosphorylated glycogen synthase (GYS1) was significantly increased following MCAO, whereas EE markedly reversed this increase in GYS1 protein levels (*p* < 0.01, Figure 2H). Importantly, the GYS1 antibody used in this study specifically recognizes the phosphorylated (inactive) form of glycogen synthase, indicating that cerebral ischemia promotes inhibitory phosphorylation of glycogen synthase, thereby suppressing glycogen synthesis, whereas EE alleviates this inhibitory modification and partially restores glycogen synthetic capacity after ischemic injury. Consistent with these molecular analyses, GYS1 immunofluorescence revealed an enhanced GYS1 signal in the MCAO group, which was significantly attenuated by EE exposure (*p* < 0.001, Figure 2A,E). Together, these findings suggest that EE suppresses excessive glycogen breakdown and relieves inhibitory constraints on glycogen synthesis following ischemic insult.

Biochemical analysis of glycogen content showed a substantial decrease in peri-infarct region glycogen levels following MCAO, whereas EE significantly increased glycogen content compared with the MCAO group (*p* < 0.01, Figure 2I). LC-MS quantification of UDP-glucose (UDPG), the direct precursor for glycogen synthesis, revealed that MCAO significantly reduced UDPG levels, whereas EE significantly elevated UDPG abundance (*p* < 0.05, Figure 2J), indicating that EE not only modulates glycogen synthase activity but also restores the substrate availability required for glycogen synthesis. Meanwhile, the lipid peroxidation marker MDA was markedly elevated in the MCAO group but significantly reduced following EE intervention (*p* < 0.001, Figure 2K), further indicating that improvement of glycogen metabolic remodeling is accompanied by attenuation of oxidative stress and ferroptotic injury.

Together, these results suggest that EE promotes adaptive glycogen metabolic reprogramming by enhancing glycogen synthesis, suppressing excessive glycogen degradation, restoring glycogen reserves, and concomitantly alleviating oxidative stress after CIRI.

### 3.3. Enriched Environment Suppresses CIRI-Induced Ferroptosis Associated with Activation of the SIRT1/AKT/GSK3β Signaling Axis

**Figure 3:**
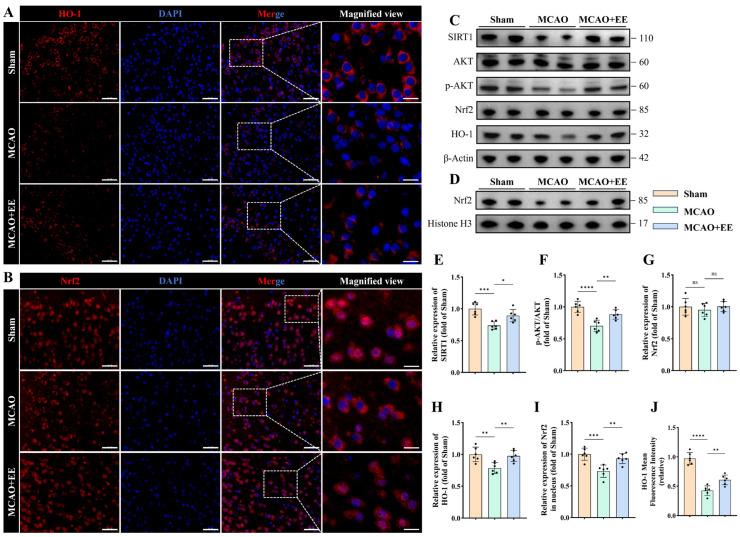
EE was associated with the SIRT1/AKT/GSK3β pathway and may contribute to Nrf2/HO-1 signaling to suppress ferroptosis after CIRI. (**A**) Representative immunofluorescence images of HO-1 (red) with DAPI nuclear staining (blue); scale bar: 50 µm (main images); 16.7 µm (magnified views). (**B**) Representative immunofluorescence images of Nrf2 illustrate its accumulation in neuronal nuclei and cytoplasm across different experimental groups; scale bar: 50 µm (main images); 16.7 µm (magnified views). (**C**) Representative Western blot images showing SIRT1, AKT, p-AKT, total Nrf2, and HO-1 expression in cortical tissue. (**D**) Nuclear Western blot showing levels of nuclear Nrf2 (n-Nrf2). (**E**–**H**) Quantitative analysis of SIRT1, p-AKT/AKT ratio, total Nrf2, and HO-1 expression (*n* = 6). (**I**) Quantitative analysis of nuclear Nrf2 (n-Nrf2) protein expression (*n* = 6). (**J**) Quantification of HO-1 fluorescence intensity (*n* = 6). All data are expressed as mean ± standard deviation (SD). Statistical comparisons were performed using one-way ANOVA followed by Tukey’s post hoc test. The white dashed boxes indicate the regions shown in the magnified views. ns, not significant; * *p* < 0.05, ** *p* < 0.01, *** *p* < 0.001, **** *p* < 0.0001.

To elucidate the signaling mechanisms by which EE mitigates ferroptosis following cerebral ischemia–reperfusion injury (CIRI), the expression of SIRT1 and activation status of its downstream AKT pathway [38] were first examined. Western blot analysis revealed a significant reduction in SIRT1 protein levels in the MCAO group compared with the Sham group, whereas EE markedly restored SIRT1 expression (*p* < 0.05, Figure 3C,E). In parallel, the p-AKT/AKT ratio was significantly decreased after MCAO, indicating suppressed AKT activity; EE intervention significantly elevated the p-AKT/AKT ratio (*p* < 0.01, Figure 3F), suggesting reactivation of the SIRT1/AKT signaling pathway [39].

The antioxidant nuclear factor erythroid 2–related factor 2 (Nrf2)/heme oxygenase-1 (HO-1) pathway was subsequently evaluated. At the total protein level, Nrf2 expression was slightly reduced in the MCAO group compared with the Sham group, but the difference did not reach statistical significance, and EE did not significantly alter total Nrf2 abundance (*p* > 0.05, Figure 3G). However, nuclear protein analysis demonstrated a marked reduction in nuclear Nrf2 (n-Nrf2) in the MCAO group, whereas EE significantly increased n-Nrf2 abundance (*p* < 0.01, Figure 3D,I), indicating that EE may contribute to Nrf2 nuclear translocation rather than altering its overall expression. Consistently, immunofluorescence staining further confirmed reduced nuclear localization of Nrf2 after MCAO, which was markedly restored by EE intervention (Figure 3B), providing cellular-level evidence for enhanced Nrf2 nuclear translocation.

Consistent with Nrf2 activation, the expression of its downstream antioxidant effector HO-1 was significantly downregulated after MCAO but robustly restored by EE intervention (*p* < 0.01, Figure 3C,H), suggesting that EE may contribute to antioxidant defense capacity in ischemic brain tissue [40]. At the cellular level, HO-1 immunofluorescence further corroborated these findings. MCAO markedly diminished HO-1 fluorescence intensity in neurons (Figure 3A), whereas EE significantly enhanced HO-1 signal intensity, with quantitative analysis confirming significantly higher HO-1 expression in the MCAO + EE group compared with the MCAO group (*p* < 0.01, Figure 3A,J).

In conclusion, these findings suggest that EE is associated with the SIRT1/AKT/GSK3β axis, promotes Nrf2 nuclear translocation and HO-1 expression, thereby enhancing antioxidant capacity and suppressing ferroptosis following CIRI.

### 3.4. Pharmacological Inhibition of SIRT1 Abolishes the Anti-Ferroptotic and Cerebroprotection Effects of Enriched Environment After CIRI

**Figure 4:**
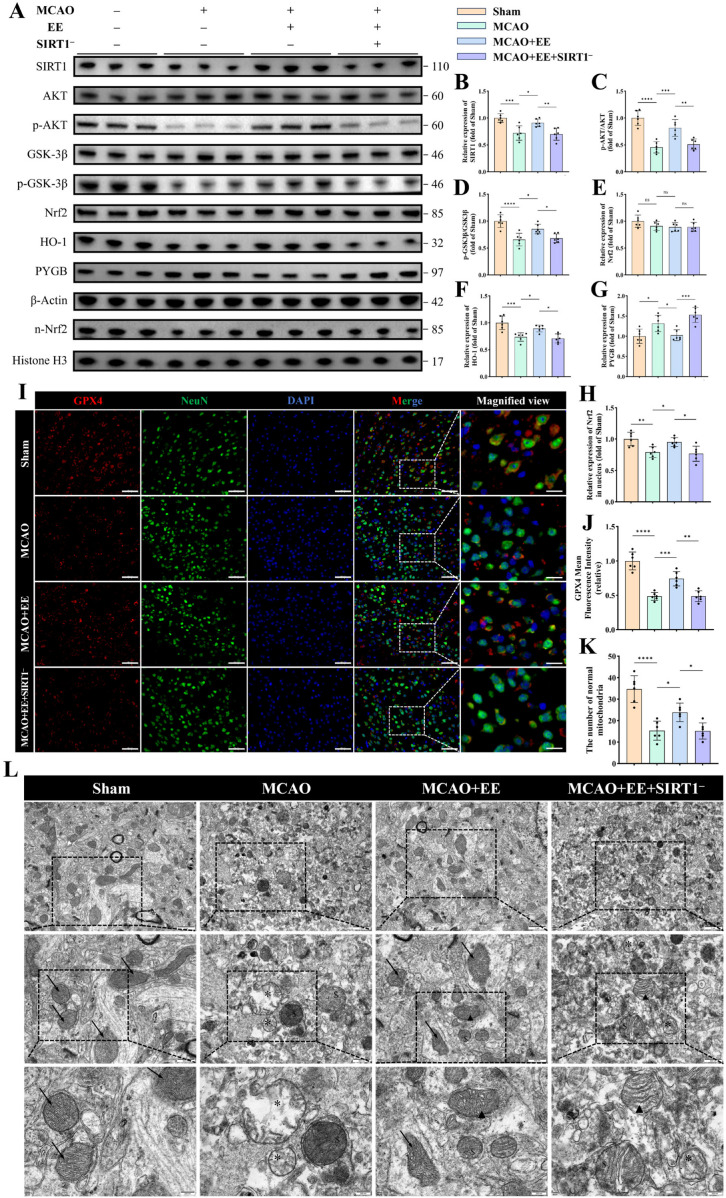
SIRT1 inhibition reverses the protective effects of EE on ferroptosis and mitochondrial integrity after CIRI. (**A**–**D**) Representative Western blot bands and quantitative analysis of proteins in the SIRT1/AKT/GSK3β signaling pathway, including SIRT1, p-AKT/AKT, and p-GSK3β/GSK3β (*n* = 6). (**E**–**G**) Representative Western blot images and corresponding quantitative data for the Nrf2/HO-1 axis and the glycogenolytic enzyme PYGB (*n* = 6). (**H**) Quantitative analysis of nuclear Nrf2 (n-Nrf2) protein expression determined by Western blotting (*n* = 6). (**I**) Representative immunofluorescence images showing the subcellular distribution of GPX4 in peri-infarct region neurons; scale bar: 50 µm (main images); 16.7 µm (magnified views). (**J**) Quantification of GPX4 immunofluorescence intensity in peri-infarct region neurons (*n* = 6). (**K**) Statistical image of normal mitochondria under transmission electron microscopy (*n* = 6). (**L**) Representative transmission electron microscopy images showing mitochondrial ultrastructural changes in cortical neurons from each group; scale bar: 1 µm, 500 nm and 200 nm. Black arrows: healthy mitochondria with intact cristae and matrix; $: swollen mitochondria with disorganized or absent cristae; *: mitochondria exhibiting decreased electron density of the matrix or vacuolization; triangles: mitochondria showing swelling of cristae or matrix. All data are presented as mean ± SD. Group comparisons were performed using one-way ANOVA followed by Tukey’s post hoc test. The dashed boxes indicate the regions shown in the magnified views. ns, not significant; * *p* < 0.05, ** *p* < 0.01, *** *p* < 0.001, **** *p* < 0.0001.

To assess whether SIRT1 is involved in the ferroptosis-related and cerebroprotective effects of EE, a selective pharmacological SIRT1 inhibitor, EX-527 (Selisistat) [19,41], was administered, and the consequent changes in key signaling proteins and cellular structures were systematically examined. Western blot analysis showed that EE markedly upregulated SIRT1 expression, enhanced AKT phosphorylation, and increased the p-GSK3β/GSK3β ratio (*p* < 0.05, Figure 4A–D), whereas these EE-induced effects were all significantly abolished by SIRT1 inhibition (*p* < 0.05, Figure 4A–D). Likewise, the antioxidant Nrf2/HO-1 axis exhibited parallel changes: total Nrf2 protein levels did not differ significantly among groups (*p* > 0.05, Figure 4E), whereas nuclear Nrf2 (n-Nrf2) was markedly reduced following MCAO, restored by EE, and subsequently decreased again after SIRT1 inhibition (*p* < 0.05, Figure 4H). Consistently, HO-1 expression was diminished after MCAO, markedly restored by EE, and significantly suppressed again upon SIRT1 inhibition (*p* < 0.05, Figure 4F). In addition, the ferroptosis-associated glycogenolytic enzyme PYGB was elevated after MCAO, suppressed by EE, and re-elevated following SIRT1 inhibition (*p* < 0.05, Figure 4A,G), further indicating that SIRT1 is required for EE-mediated regulation of glycogen metabolism under ischemic stress.

At the cellular level, immunofluorescence analysis of GPX4 further supported the SIRT1 dependency of EE-mediated ferroptosis suppression: GPX4 immunoreactivity in peri-infarct region neurons was reduced after MCAO, consistently increased following EE exposure, and significantly attenuated again after SIRT1 blockade, as confirmed by the corresponding quantitative analysis (*p* < 0.01, Figure 4I,J).

Ultrastructural assessment by transmission electron microscopy (TEM) further illustrated the functional consequences of these molecular alterations. Neurons in the MCAO group exhibited pronounced ferroptosis-associated mitochondrial abnormalities, characterized by severe mitochondrial swelling, loss of membrane integrity, disrupted or absent cristae, and decreased matrix density, whereas EE markedly preserved mitochondrial ultrastructure in ischemic neurons. Importantly, the protective effect of EE on mitochondrial integrity was largely abolished by SIRT1 inhibition, resulting in mitochondrial damage comparable to that observed in the MCAO group (Figure 4K,L).

Taken together, these results suggest that SIRT1 contributes to EE-associated activation of the AKT/GSK3β/Nrf2 signaling cascade, maintenance of redox homeostasis, regulation of glycogen metabolism, modulation of ferroptosis-related processes, and preservation of mitochondrial integrity following CIRI.

### 3.5. SIRT1 Overexpression Restores Glycogen Metabolic Homeostasis and Attenuates Ferroptosis in Ischemic–Hypoxic SH-SY5Y

To further investigate the role of SIRT1 in glycogen metabolism and ferroptosis-related processes under ischemic–hypoxic conditions, in vitro experiments were performed using SH-SY5Y cells. Given that in vivo results may be influenced by multiple cell types and systemic factors, this approach provides a more controlled experimental setting. An SH-SY5Y cell-based SIRT1 overexpression model was generated and subjected to oxygen–glucose deprivation/reoxygenation (OGD/R). This model was used to examine the effects of SIRT1 overexpression on glycogen metabolism and ferroptosis-related changes, and to assess whether these cellular responses are consistent with the protective effects observed following EE intervention in vivo.

**Figure 5:**
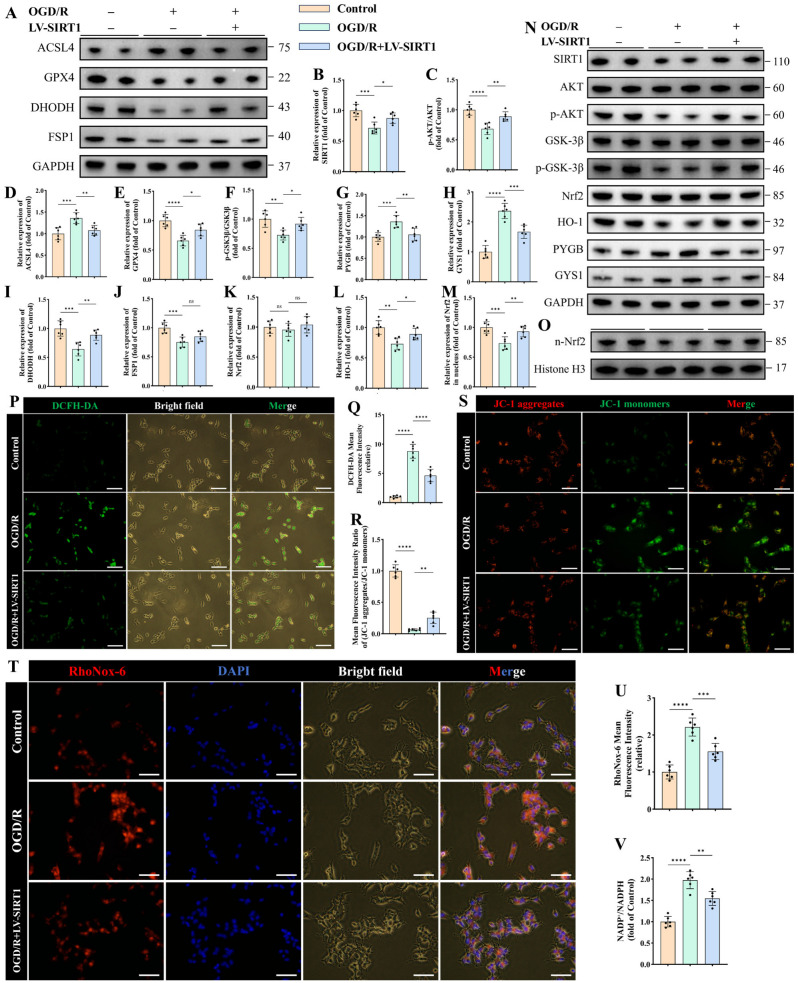
SIRT1 overexpression restores glycogen metabolic homeostasis and attenuates ferroptosis in ischemic–hypoxic neurons. (**A**,**D**,**E**) Representative Western blot bands and quantitative analysis of ferroptosis-related proteins ACSL4 and GPX4 (*n* = 6). (**B**,**C**,**F**) Quantitative analysis of SIRT1, p-AKT/AKT, p-GSK-3β/GSK-3β, Nrf2, HO-1, PYGB and GYS1 (*n* = 6). (**G**,**H**,**K**,**L**) Quantitative analysis of PYGB, GYS1, Nrf2 and HO-1 (*n* = 6). (**I**,**J**) Quantitative analysis of DHODH and FSP1 expression (*n* = 6). (**N**) Representative Western blot bands showing the expression of proteins associated with the SIRT1/AKT/GSK-3β signaling pathway and ferroptosis regulation. (**M**,**O**) Representative Western blot bands and expression levels of nuclear Nrf2 (n-Nrf2), with Histone H3 used as the nuclear loading control. (**P**,**Q**) Representative DCFH-DA fluorescence images showing ROS levels and quantification of intracellular levels (*n* = 6), bar: 50 μm. (**R**) Quantification of JC-1 red/green fluorescence ratio to evaluate mitochondrial membrane potential (*n* = 6). (**S**) Representative JC-1 fluorescence images showing mitochondrial membrane potential, with red JC-1 aggregates indicating high membrane potential and green JC-1 monomers indicating depolarization, bar: 50 μm. (**T**) Representative RhoNOX-6 fluorescence images showing intracellular Fe^2+^ with DAPI counter-staining for nuclei, bar: 50 μm. (**U**) Quantification of intracellular Fe^2+^ levels measured by RhoNOX-6 fluorescence (*n* = 6). (**V**) Quantification of intracellular NADP^+^/NADPH ratio in cells (*n* = 6). All data are presented as mean ± SD. Statistical comparisons were performed using one-way ANOVA followed by Tukey’s post hoc test. ns, not significant; * *p* < 0.05, ** *p* < 0.01, *** *p* < 0.001, **** *p* < 0.0001.

Under OGD/R conditions, SIRT1 overexpression in SH-SY5Y cells markedly attenuated ferroptosis-related changes. This protective effect was first reflected at the phenotypic level by a significant reduction in the ferroptosis-promoting enzyme ACSL4 (*p* < 0.01, Figure 5A,D). Concomitantly, other ferroptosis-related regulators, including GPX4, DHODH, and FSP1, were assessed (Figure 5E,I,J). Notably, SIRT1 overexpression significantly restored GPX4 (*p* < 0.05, Figure 5E) and the mitochondrial inner membrane-associated enzyme DHODH expression (*p* < 0.01, Figure 5I), whereas the outer mitochondrial membrane ferroptosis suppressor FSP1 showed no significant change (*p* > 0.05, Figure 5J), suggesting that SIRT1-mediated ferroptosis regulation is predominantly dependent on the GPX4- and DHODH-associated pathways rather than the FSP1 axis.

Mechanistically, Western blot analysis demonstrated that SIRT1 overexpression significantly increased cellular SIRT1 abundance, enhanced AKT phosphorylation, and elevated the p-GSK-3β/GSK-3β ratio (*p* < 0.05, Figure 5B,C,F), indicating reactivation of the SIRT1/AKT/GSK-3β signaling cascade. Regarding glycogen metabolism, SIRT1 overexpression significantly reduced the expression of the glycogenolytic enzyme PYGB (*p* < 0.01, Figure 5G), while glycogen synthase GYS1 showed the opposite trend (*p* < 0.001, Figure 5H), indicating attenuation of ischemia-induced excessive glycogen breakdown and restoration of glycogen metabolic homeostasis.

To further evaluate intracellular redox metabolic status, the NADP^+^/NADPH ratio was measured using a commercial assay kit following the respective treatments in SH-SY5Y cells. The results showed that OGD/R markedly increased the NADP^+^/NADPH ratio (*p* < 0.0001, Figure 5V), indicating impaired cellular redox balance, whereas SIRT1 overexpression significantly reversed this abnormal elevation (*p* < 0.01, Figure 5V), suggesting restoration of reducing capacity and redox homeostasis.

In parallel, activation of antioxidant signaling was observed. Nuclear protein assays revealed that SIRT1 overexpression markedly enhanced Nrf2 nuclear translocation, as evidenced by increased nuclear Nrf2 levels normalized to Histone H3 (*p* < 0.01, Figure 5M,O), with no significant difference in the expression of Nrf2 in total protein among the different groups (*p* > 0.05, Figure 5K). Consistently, the expression of the downstream antioxidant effector HO-1 was significantly upregulated (*p* < 0.05, Figure 5L), closely mirroring the effects of EE intervention observed in vivo and further supporting SIRT1 as a critical mediator of EE-associated neuroprotection.

Functional fluorescent assays provided additional cellular-level evidence. DCFH-DA staining showed pronounced ROS accumulation in OGD/R-treated neurons, which was dramatically attenuated by SIRT1 overexpression (*p* < 0.0001, Figure 5P,Q). RhoNOX-6 staining demonstrated robust intracellular Fe^2+^ accumulation following OGD/R, whereas SIRT1 overexpression significantly reduced Fe^2+^ fluorescence intensity (*p* < 0.001, Figure 5T,U). JC-1 staining further indicated that OGD/R induced a collapse of mitochondrial membrane potential, reflected by a shift from red JC-1 aggregates to green monomers, while SIRT1 overexpression partially restored mitochondrial membrane potential, as shown by an increased red/green fluorescence ratio (*p* < 0.01, Figure 5R,S).

Taken together, these findings indicate that upregulation of SIRT1 in SH-SY5Y cells confers a protective effect under ischemic–hypoxic conditions. Specifically, SIRT1 overexpression was associated with improved glycogen metabolic balance, attenuation of ferroptosis-related processes, enhancement of antioxidant capacity, and preservation of mitochondrial function. Notably, these in vitro observations are consistent with our in vivo findings, supporting the notion that SIRT1 activation may represent a key mechanism underlying the neuroprotective effects observed following enriched environment intervention. In particular, SIRT1 upregulation in OGD/R-treated SH-SY5Y cells exerted comparable protective effects, including modulation of ferroptosis-associated pathways and normalization of key enzymes involved in glycogen metabolism.

### 3.6. SIRT1-Driven Glycogen Metabolic Reprogramming Modulates Ferroptosis-Associated Pathways in Ischemic–Hypoxic Neurons

To determine whether glycogen metabolic reprogramming is functionally required for the attenuation of ferroptosis under ischemic–hypoxic conditions, we evaluated ROS production, ferroptosis-related proteins, intracellular Fe^2+^ accumulation, and biochemical correlates across four groups in the OGD/R model: Control, OGD/R, OGD/R + LV-SIRT1, and OGD/R + LV-SIRT1 + MZ-101. Importantly, the OGD/R + LV-SIRT1 + MZ-101 group consisted of SIRT1-overexpressing SH-SY5Y cells subjected to OGD/R and subsequently treated with the glycogen synthase inhibitor MZ-101, direct enzymatic inhibition, allowing assessment of whether disruption of glycogen synthesis attenuates the protective effects associated with SIRT1 overexpression.

**Figure 6:**
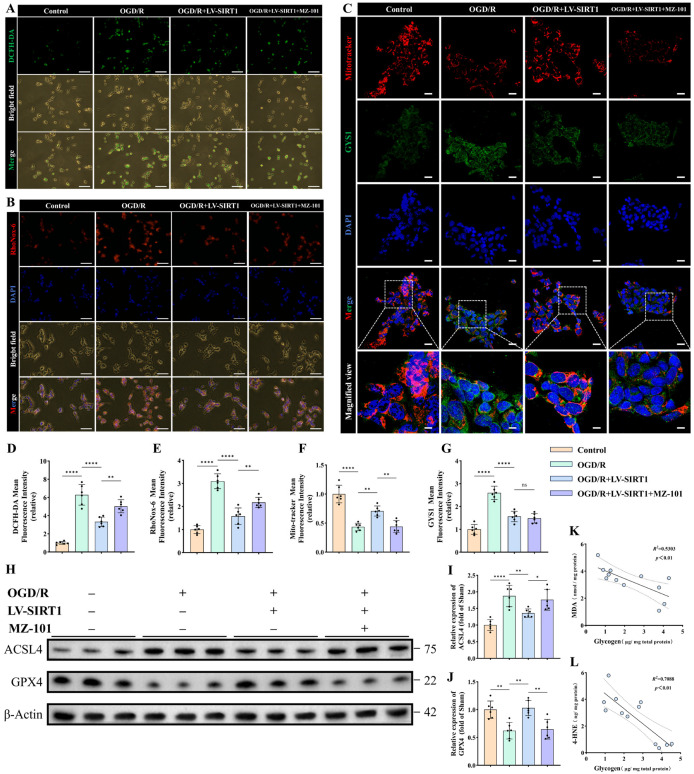
SIRT1-driven glycogen metabolic reprogramming modulates ferroptosis-associated pathways in ischemic–hypoxic neurons. (**A**,**D**) Representative DCFH-DA fluorescence images and quantification of intracellular ROS levels in each group (*n* = 6); scale bar: 50 µm. (**B**,**E**) Representative RhoNOX-6 fluorescence images showing intracellular Fe^2+^ levels (red) with DAPI nuclear counterstaining (blue), along with quantification of mean fluorescence intensity in each group (*n* = 6); scale bar: 50 µm. (**C**) Representative immunofluorescence images showing the subcellular distribution of Mito Tracker (red) and GYS1 (green) in each group of SH-SY5Y cells. The white dashed boxes indicate the regions shown in the magnified views; scale bar: 20 µm (main images); 6.7 µm (magnified views). (**F**,**G**) Quantification of fluorescence intensity of MitoTracker (red) and GYS1 (green) in each of the experimental groups (*n* = 6). (**H**–**J**) Representative Western blot bands and quantitative analysis of ACSL4 and GPX4 expression, with β-Actin used as the loading control (*n* = 6). (**K**,**L**) Pearson correlation analysis between intracellular glycogen content and lipid peroxidation markers, including MDA and 4-HNE. All data are presented as mean ± SD. Group comparisons were performed using one-way ANOVA followed by Tukey’s post hoc test. Correlation analyses were conducted using Pearson correlation testing. ns, not significant; * *p* < 0.05, ** *p* < 0.01, **** *p* < 0.0001.

DCFH-DA fluorescence imaging revealed a marked increase in green ROS fluorescence in the OGD/R group, whereas SIRT1 overexpression consistently suppressed ROS accumulation. Notably, this antioxidant effect was significantly attenuated by MZ-101 treatment, with ROS levels partially rebounding compared with the OGD/R + LV-SIRT1 group (Figure 6A). Quantitative analysis confirmed that ROS levels were significantly elevated following OGD/R, substantially suppressed by SIRT1 overexpression (*p* < 0.0001, Figure 6D), and partially reversed upon MZ-101 treatment (*p* < 0.01, Figure 6D), suggesting that inhibition of glycogen synthesis compromises the antioxidant capacity and the regulation of ferroptosis-related mechanisms conferred by SIRT1.

Intracellular Fe^2+^ staining using RhoNox-6 showed a marked increase in red fluorescence in the OGD/R group, indicating substantial iron accumulation (Figure 6B). In contrast, SIRT1 overexpression significantly reduced Fe^2+^ accumulation following OGD/R, whereas this inhibitory effect was significantly attenuated by MZ-101 treatment, resulting in a rebound of intracellular Fe^2+^ levels compared with the LV-SIRT1 group (Figure 6B). Quantitative analysis confirmed that Fe^2+^ levels were significantly elevated after OGD/R, substantially decreased by LV-SIRT1 (*p* < 0.0001, Figure 6E), and significantly reversed upon MZ-101 treatment (*p* < 0.01, Figure 6E), suggesting that disruption of glycogen synthesis may attenuate the reduction in iron accumulation induced by SIRT1 overexpression [42].

To further explore the subcellular basis underlying this effect, we performed co-immunofluorescence staining of mitochondria using Mito-Tracker Red and glycogen synthase 1 (GYS1) using a green fluorescent marker. As shown in Figure 6C, OGD/R exposure resulted in a marked reduction in mitochondrial fluorescence intensity, accompanied by an increase in GYS1 signal (*p* < 0.0001, Figure 6F,G), suggesting impaired mitochondrial integrity and enhanced inhibitory phosphorylation of glycogen synthase under ischemic stress. Notably, SIRT1 overexpression significantly restored mitochondrial fluorescence (*p* < 0.01, Figure 6F), indicating improved mitochondrial viability, while simultaneously reducing GYS1 fluorescence intensity (*p* < 0.0001, Figure 6G). These findings suggest that SIRT1 alleviates OGD/R-induced mitochondrial damage and suppresses the inhibitory phosphorylation of GYS1, thereby promoting glycogen metabolic homeostasis. In contrast, treatment with MZ-101 markedly attenuated the protective effects of SIRT1 on mitochondrial integrity, as evidenced by reduced MitoTracker fluorescence compared with the OGD/R + LV-SIRT1 group (*p* < 0.01, Figure 6F). Interestingly, MZ-101 administration did not significantly alter GYS1 fluorescence intensity (*p* > 0.05, Figure 6G), despite effectively impairing glycogen synthesis. This observation implies that MZ-101 primarily inhibits glycogen synthesis through direct enzymatic inhibition rather than by altering the phosphorylation status of GYS1. Collectively, these results further support that SIRT1-mediated neuroprotection is closely associated with glycogen metabolic regulation, and pharmacological disruption of glycogen synthesis diminishes its beneficial effects on mitochondrial function.

Western blot analyses of ferroptosis-related proteins showed that OGD/R dramatically upregulated the ferroptosis-promoting enzyme ACSL4 and significantly downregulated the ferroptosis-suppressing enzyme GPX4 (*p* < 0.01, Figure 6H–J). Quantitative analysis showed that ACSL4 expression was significantly increased in the OGD/R group (*p* < 0.0001, Figure 6I), whereas SIRT1 overexpression markedly reduced ACSL4 levels (*p* < 0.01, Figure 6I). Notably, this inhibitory effect of SIRT1 on ACSL4 expression was significantly attenuated by MZ-101 treatment (*p* < 0.05, Figure 6I), indicating that blockade of glycogen synthesis impairs the regulation of ferroptosis-related mechanisms mediated by SIRT1-driven metabolic reprogramming. Conversely, GPX4 expression was significantly decreased following OGD/R, while LV-SIRT1 markedly restored GPX4 levels (*p* < 0.01, Figure 6H,J). In contrast, MZ-101 markedly blunted the SIRT1-induced recovery of GPX4 expression (*p* < 0.01, Figure 6H,J), further supporting the notion that the protective regulation of GPX4 by SIRT1 is dependent on intact glycogen metabolic reprogramming.

Pearson correlation analyses revealed a strong significant association between glycogen metabolism and lipid peroxidation. Specifically, glycogen content was negatively correlated with lipid peroxidation markers: glycogen levels showed a significant inverse correlation with MDA (R^2^ = 0.5303, *p* < 0.01, Figure 6K) and an even stronger negative correlation with 4-HNE levels (R^2^ = 0.7088, *p*< 0.01, Figure 6L). These findings indicate that higher glycogen reserves are tightly associated with reduced lipid peroxidation and attenuated ferroptotic injury, supporting a functional linkage between glycogen metabolic status and ferroptosis-related oxidative damage under ischemic–hypoxic conditions [43].

### 3.7. Enriched Environment Is Associated with Improvements in Neurological Outcomes After CIRI Through the SIRT1/AKT/GSK3β Signaling Pathway In Vivo

To determine whether EE ameliorates motor dysfunction after CIRI in a SIRT1/AKT/GSK3β pathway-dependent manner, locomotor behavior, infarct severity, and neuronal death were systematically evaluated across experimental groups.

**Figure 7:**
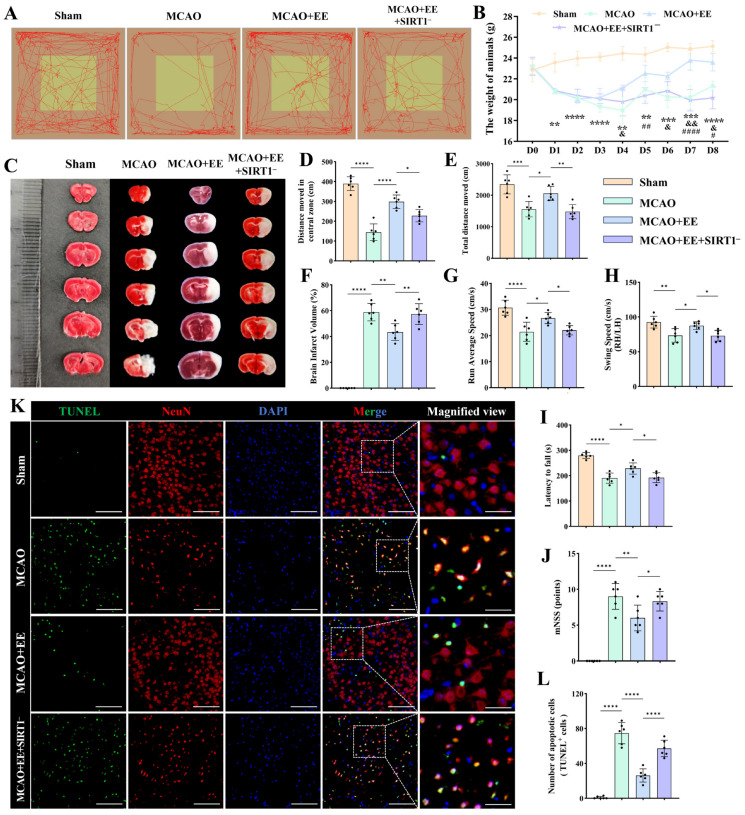
Enriched environment may improve neurological outcomes after CIRI through the SIRT1/AKT/GSK3β signaling pathway in vivo. (**A**) Representative open-field tracking maps showing spontaneous activity patterns across groups. (**B**) Body-weight changes over time following surgery. * indicates the comparison between MCAO and Sham groups; & indicates the comparison between MCAO and MCAO + EE groups; # indicates the comparison between MCAO + EE and MCAO + EE + SIRT1^−^ groups. The number of corresponding symbols represents the significance of the p-value and the difference, (*n* = 6). (**C**) Representative TTC-stained brain sections showing infarct areas in each group. (**D**) Quantification of center-zone distance traveled in the open-field test (*n* = 6). (**E**) Quantification of total distance traveled in the open-field test (*n* = 6). (**F**) Quantification of infarct volume based on TTC staining (*n* = 6). (**G**,**H**) Gait analysis of average running velocity and swing speed across groups (*n* = 6). (**I**) Rotarod test showing latency to fall (*n* = 6). (**J**) Modified neurological severity score (mNSS) assessment across groups (*n* = 6). (**K**,**L**) Representative TUNEL/NeuN/DAPI fluorescence images and quantification of TUNEL-positive neurons for assessment of neuronal cell death (*n* = 6). The white dashed boxes indicate the regions shown in the magnified views; scale bar: 50 µm (main images); 16.7 µm (magnified views). All data are presented as mean ± SD. Differences in body weight across time points and groups were evaluated using two-way ANOVA followed by Tukey’s post hoc test for multiple comparisons. Statistical analysis was performed using one-way ANOVA followed by Tukey’s post hoc test, for all other datasets involving comparisons among multiple groups. * *p* < 0.05, ** *p* < 0.01, *** *p* < 0.001, **** *p* < 0.0001, & *p* < 0.05, && *p* < 0.01, # *p* < 0.05, ## *p* < 0.01, #### *p* < 0.0001.

In terms of spontaneous locomotion, open-field tracking revealed a marked reduction in exploratory range in the MCAO group, whereas EE significantly increased activity dispersion and spatial exploration. In contrast, SIRT1 deficiency greatly blunted this EE-induced improvement (Figure 7A), indicating that SIRT1 is required for EE-mediated recovery of spontaneous locomotor activity. The distance traveled in the center area of the open field was markedly reduced in the MCAO group but significantly increased following EE intervention (*p* < 0.0001, Figure 7A,D), reflecting improved exploratory behavior and reduced anxiety-like phenotype. This improvement was significantly attenuated in SIRT1-deficient mice (*p* < 0.05, Figure 7A,D), indicating SIRT1-dependent regulation of emotional and exploratory behavior. Consistent results were observed in total traveling distance, with EE promoting overall locomotor activity recovery, whereas SIRT1 deficiency substantially limited this effect (*p* < 0.05, Figure 7A,E).

Meanwhile, gait assessments revealed that running velocity was significantly slowed in MCAO mice (*p* < 0.0001, Figure 7G), whereas EE significantly improved this parameter (*p* < 0.05, Figure 7G), indicating enhanced motor coordination and strength. In contrast, SIRT1-deficient mice exhibited insufficient recovery (*p* < 0.05, Figure 7G). Swing speed showed a similar pattern (*p* < 0.05, Figure 7H), further supporting that EE improves locomotor coordination through a SIRT1-dependent mechanism. In the rotarod test, MCAO mice showed a significantly shortened latency to fall (*p* < 0.0001, Figure 7I), whereas EE dramatically prolonged retention time (*p* < 0.05, Figure 7I). In contrast, SIRT1 deficiency markedly impaired the recovery of balance performance (*p* < 0.05, Figure 7I), indicating that EE-mediated motor learning and coordination require intact SIRT1 signaling. Similar results were obtained with the Modified neurological severity score (*p* < 0.05, Figure 7J).

Body-weight monitoring showed that MCAO mice exhibited a continuous decline in body weight during the early ischemia–reperfusion phase (day 1–4), whereas EE significantly promoted weight recovery starting from day 4. In contrast, SIRT1-deficient mice displayed persistently impaired weight regain following CIRI (*p* < 0.05, Figure 7B), suggesting compromised systemic recovery in the absence of SIRT1 signaling.

TTC staining demonstrated a large infarct area in MCAO mice, whereas EE markedly reduced infarct volume (*p* < 0.01, Figure 7C,F), indicating substantial cerebroprotection. Notably, the loss of SIRT1 significantly diminished the infarct-sparing effect of EE (*p* < 0.01, Figure 7C,F).

Neuronal cell death was evaluated using TUNEL staining. MCAO mice exhibited a pronounced increase in TUNEL-positive neurons, indicating enhanced neuronal cell death, whereas EE reduced the number of TUNEL-positive neurons (*p* < 0.0001, Figure 7K,L), demonstrating strong neuroprotective efficacy. Notably, pharmacological inhibition of SIRT1 significantly attenuated the protective effect of EE, linked to increased neuronal cell death compared with the MCAO + EE group (*p* < 0.0001, Figure 7K,L).

Collectively, these data suggest that EE is associated with improved neurological function, reduced infarct burden, and decreased cell death following CIRI, and that these effects are associated with SIRT1/AKT/GSK3β signaling.

## 4. Discussion

In the present study, we employed both in vivo MCAO/R and in vitro OGD/R models to comprehensively investigate the cerebroprotection mechanisms by which EE mitigates cerebral ischemia–reperfusion injury. Our findings suggest that enriched environment (EE) exerts robust protective effects that are closely associated with the activation of the SIRT1/AKT/GSK3β signaling axis, accompanied by coordinated changes in glycogen metabolism, ferroptosis-related pathways, and mitochondrial function. These effects were largely attenuated by pharmacological inhibition of SIRT1, supporting a central role for SIRT1 in mediating EE-induced protection. Importantly, this integrated protective profile highlights a previously underappreciated metabolic dimension of EE-mediated cerebroprotection. Mechanistically, EE promotes SIRT1 activation, which is linked to enhanced AKT phosphorylation and inhibitory regulation of GSK3β, which in turn facilitates adaptive remodeling of neuronal glycogen metabolism. This coordination may link external environmental stimulation to intracellular metabolic responses. These effects are associated with reduced lipid peroxidation, attenuation of ferroptotic damage, preservation of mitochondrial homeostasis, and activation of the Nrf2/HO-1 antioxidant pathway [44], ultimately improving neurological function after CIRI.

Although EE produced robust protective effects in the present study, the specific components responsible for these effects remain difficult to disentangle. Previous studies have suggested that EE is composed of multiple intervention elements, such as physical and social components, which may differentially or synergistically modulate behavioral performance and neurobiological responses, rather than producing uniform effects across experimental groups [45]. Therefore, the present findings should be interpreted as reflecting the integrated effects of EE as a composite intervention, and future studies employing more controlled paradigms will be required to dissect the relative contribution of individual components. Importantly, the intervention paradigm used in this study may have translational relevance in the context of current stroke treatment strategies. Enriched environment (EE) represents a non-invasive, post-insult intervention that can be implemented after reperfusion, making it inherently compatible with clinical recanalization approaches such as thrombolysis or mechanical thrombectomy. This aligns well with recent recommendations from the Stroke Treatment Academic Industry Roundtable [46], which emphasize the importance of developing adjunctive therapies that can be applied in combination with reperfusion therapies to enhance neurological recovery. In this context, EE-induced metabolic and redox modulation may represent a promising complementary strategy to improve post-stroke outcomes.

Ischemia and subsequent reperfusion impose profound metabolic and oxidative challenges on neuronal tissue. The abrupt restoration of oxygen supply following prolonged ischemia is associated with excessive ROS production, mitochondrial membrane depolarization, lipid peroxidation, and catastrophic bioenergetic collapse [47]. Recent evidence underscores that ferroptosis, a regulated cell death modality dependent on iron-driven lipid peroxidation, plays a crucial role in determining neuronal viability in the reperfusion phase. In line with this emerging paradigm, our data indicate that ferroptotic signaling is prominently activated during CIRI. Consistent with prior reports [48], marked increases in ACSL4 expression, GPX4 depletion, and elevated levels of lipid peroxides such as MDA and 4-HNE were observed, along with characteristic mitochondrial fragmentation in CIRI models. These pathological changes were robustly reversed by EE, which significantly enhanced GPX4 expression, reduced lipid peroxidation, and preserved mitochondrial morphology, thereby supporting a role for EE as an effective modulator of ferroptotic vulnerability [49]. While these findings are consistent with ferroptosis modulation, it should be noted that markers such as ACSL4 and GPX4, although widely used, are not entirely specific to ferroptosis and may also be altered in other forms of oxidative stress-related cell death. Given the complexity of ischemia–reperfusion injury, where multiple cell death pathways coexist and interact, the attribution of the observed protective effects exclusively to ferroptosis should be interpreted with caution. Future studies incorporating ferroptosis-specific rescue strategies or pathway-targeted interventions will be necessary to further substantiate this mechanistic link.

Remarkably, the regulation of ferroptosis-related mechanisms by EE was found to be closely associated with glycogen metabolic remodeling, an underexplored yet biologically significant component of cerebral energy metabolism [50]. This observation provides a novel metabolic perspective on how EE translates into cellular stress resistance. Unlike glycolysis and oxidative phosphorylation, brain glycogen metabolism has historically received limited attention in stroke research. Nevertheless, glycogen functions as a critical energy reserve in astrocytes and neurons, particularly under conditions of acute metabolic stress. In the present models, ischemic injury was accompanied by a pronounced upregulation of the glycogen phosphorylase PYGB, indicative of excessive glycogen breakdown, whereas EE intervention and neuronal SIRT1 overexpression significantly suppressed PYGB expression while increasing overall glycogen content in both peri-infarct brain tissue and OGD/R-treated neurons. This pattern suggests that EE does not simply enhance overall metabolic activity, but rather reprograms glycogen handling toward an energy-conserving and stress-adaptive state. These findings suggest that EE promotes adaptive glycogen metabolic reprogramming characterized by enhanced glycogen synthesis and restrained glycogenolysis, rather than indiscriminate activation of glycogen turnover [51]. Notably, glycogen content was inversely correlated with lipid peroxidation markers, including MDA and 4-HNE, supporting a close association between glycogen metabolic status and ferroptosis-related oxidative damage. Although the precise downstream mechanisms remain to be fully elucidated, these results indicate that glycogen availability may influence neuronal vulnerability to ferroptosis, potentially through maintaining metabolic stability and limiting lipid peroxidation stress. In this context, glycogen may function not only as an energy substrate but also as a buffer system that dampens redox fluctuations under ischemic conditions. Consistent with emerging evidence linking metabolic state to lipid redox homeostasis, these findings support the existence of a glycogen metabolism–ferroptosis axis that may serve as a critical determinant of neuronal fate following ischemic injury [52].

SIRT1 emerged as a central regulator linking EE to metabolic and oxidative homeostasis. SIRT1 is a NAD^+^-dependent deacetylase known to modulate diverse cellular processes, including oxidative stress responses, mitochondrial biogenesis, autophagy, inflammation, and multiple forms of regulated cell death [53]. By virtue of its position at the interface of energy sensing and stress adaptation, SIRT1 is well suited to mediate the pleiotropic effects of EE. In the present models, CIRI was associated with a marked reduction in SIRT1 expression, whereas EE intervention significantly enhanced SIRT1 levels, consistent with its neuroprotective role. Importantly, pharmacological inhibition of SIRT1 markedly attenuated the protective effects of EE, leading to re-emergence of ferroptosis-related alterations, increased ROS accumulation, loss of mitochondrial membrane potential, and disruption of glycogen metabolic balance. In contrast, SIRT1 upregulation promoted adaptive glycogen metabolic reprogramming characterized by enhanced glycogen synthesis and restrained glycogenolysis, accompanied by preservation of mitochondrial integrity and reduction in lipid peroxidation. These bidirectional manipulations provide compelling evidence for a causal role of SIRT1 in coordinating metabolic and anti-ferroptotic responses. Collectively, these findings identify SIRT1 as a metabolic switch that integrates energy homeostasis with resistance to ferroptotic injury under ischemic stress [54].

Mechanistically, EE was associated with enhanced AKT phosphorylation and suppressed GSK3β activity in a SIRT1-dependent manner. Although SIRT1 is a deacetylase rather than a kinase, its ability to facilitate AKT activation is supported by prior studies demonstrating its indirect regulation via upstream modulators such as PTEN and FOXO proteins [55]. The AKT/GSK3β signaling axis is widely recognized as a key regulator of neuronal stress tolerance, mitochondrial integrity, and oxidative homeostasis. Consistent with previous studies showing that inhibition of GSK3β preserves mitochondrial function and limits oxidative damage under ischemic conditions [56], EE appears to favor a signaling state conducive to neuronal survival and reduced ferroptotic stress. Through this axis, EE seems to convert external stimulation into intracellular signals that promote energy storage and suppress oxidative injury. In addition to its role in glycogen metabolism, GSK3β has been increasingly recognized as a central regulator of neuronal function and vulnerability. Previous studies have shown that GSK3 signaling is involved in enriched environment-mediated neuroplasticity [57], supporting its role as a key mediator linking environmental stimulation to neuronal adaptation. Moreover, dysregulated GSK3 activity has been implicated in early neurodegenerative processes, where enhanced interaction with voltage-gated sodium channels such as Nav1.6 contributes to increased neuronal excitability and vulnerability [58]. Importantly, emerging evidence also suggests that GSK3β may directly regulate ferroptosis sensitivity through modulation of iron homeostasis and oxidative stress pathways [59]. These findings further support the notion that GSK3β represents a critical convergence point integrating metabolic regulation, redox balance, and neuronal survival, thereby reinforcing the mechanistic framework proposed in the present study. Furthermore, EE promoted nuclear translocation of Nrf2 and increased expression of its downstream effector HO-1. These effects were abolished by pharmacological inhibition of SIRT1, supporting a regulatory hierarchy in which SIRT1 modulates the Nrf2-dependent antioxidant response [60]. Given the established role of Nrf2 in regulating iron handling and lipid peroxidation [61], activation of the SIRT1–Nrf2 pathway may contribute to the ferroptosis-suppressive effects of EE following ischemic injury. This layered regulation underscores the capacity of EE to engage both metabolic and redox defense systems in a coordinated manner. It should also be noted that the role of the Nrf2/HO-1 pathway in ferroptosis is complex and may not be uniformly protective. While Nrf2 activation is generally associated with enhanced antioxidant capacity and cellular defense against oxidative stress, HO-1 induction can, under certain conditions, increase intracellular free iron levels through heme degradation, potentially exacerbating lipid peroxidation and ferroptotic processes [62]. Therefore, the overall effect of Nrf2/HO-1 activation may depend on the balance between its antioxidant functions and its impact on iron metabolism. In this context, the protective role observed in the present study should be interpreted with appropriate caution, and further investigation is needed to delineate the precise conditions under which this pathway exerts beneficial versus detrimental effects.

One of the notable strengths of this study is the integration of molecular, metabolic, cellular, and behavioral analyses within the same mechanistic framework. While EE has been previously associated with improvements in synaptic plasticity, neurogenesis, and cognitive recovery [63], its role in metabolic regulation during acute CIRI has been less understood. By demonstrating that EE directly reshapes neuronal energy metabolism and ferroptosis sensitivity, our work expands the mechanistic repertoire of EE beyond classical neuroplasticity paradigms. Behavioral data, notably improved rotarod performance, enhanced gait parameters, and increased locomotor activity, suggest that the beneficial effects of EE extend beyond cellular protection to functional recovery [64]. Importantly, inhibition of SIRT1 substantially reversed these improvements, suggesting that behavioral restoration is contingent upon metabolic and anti-ferroptotic mechanisms. This linkage between intracellular metabolic stability and macroscopic behavioral outcome provides an important translational bridge.

This observation aligns with emerging theoretical models proposing that neurobehavioral recovery after stroke is critically dependent on the restoration of metabolic stability, which serves as a prerequisite for synaptic reorganization and network repair [65].It should be noted that the in vivo MCAO/R model reflects the integrated responses of multiple cell types within the neurovascular unit, including neurons, astrocytes, and microglia. In particular, astrocytes play a critical role in regulating neuronal energy metabolism and redox homeostasis through metabolic coupling, glycogen storage, and antioxidant support [66]. These neuron–glia interactions, especially astrocyte-mediated metabolic and antioxidative processes, may influence the observed molecular and functional outcomes and cannot be fully excluded in in vivo settings. Therefore, to further investigate the role of SIRT1 under controlled conditions, we employed an in vitro OGD/R model using SH-SY5Y cells. Given that in vivo findings may be influenced by multiple cell types and systemic factors, this complementary approach provides a simplified experimental setting. This model was used to examine the involvement of the SIRT1/AKT/GSK3β signaling pathway and glycogen metabolism-related changes in SH-SY5Y cells.

This study also sheds light on mitochondrial quality control as a downstream effector of EE-mediated protection. Mitochondrial dysfunction is both a cause and a consequence of ferroptosis, and maintaining mitochondrial integrity appears to be important for neuronal survival during ischemic stress. EE restored mitochondrial membrane potential, preserved cristae structure, reduced mitochondrial ROS output, and improved mitochondrial morphology, indicating that SIRT1 activation promotes overall mitochondrial homeostasis. These findings are consistent with the known ability of SIRT1 to regulate PGC-1α-driven mitochondrial biogenesis, modulate mitophagy programs, and influence mitochondrial protein acetylation [67]. By integrating mitochondrial biogenesis, turnover, and functional preservation, SIRT1 appears to function as a multi-tiered defender of mitochondrial stability in the context of CIRI [68].

Despite these strengths, several limitations warrant discussion. First, the duration of post-stroke assessment was limited to 7 days, primarily reflecting acute and early recovery phases. According to preclinical stroke guidelines, outcome assessment should extend to at least 3 weeks to evaluate long-term recovery [69]. Whether EE-induced effects persist into the chronic phase remains to be determined [70]. Therefore, the current results primarily reflect early-stage neuroprotective effects rather than long-term outcomes. Future studies incorporating extended observation periods will be necessary to determine the durability and translational relevance of these findings. Although our findings implicate glycogen metabolism as a key determinant of ferroptosis sensitivity, we did not conduct metabolic flux analyses or single-cell metabolic profiling to directly trace glycogen-derived carbon utilization [71]. Therefore, the precise metabolic fate of glycogen under EE conditions remains to be delineated. Such analyses could clarify whether the protective effect of glycogen is mediated through increased NADPH generation, enhanced substrate availability for antioxidant pathways [72], or indirect stabilization of mitochondrial bioenergetics. Second, although EE markedly increased SIRT1 expression, the upstream triggers through which EE modulates SIRT1 remain unclear. Potential mechanisms include alterations in NAD^+^ biosynthesis, AMPK activation, circadian entrainment, and epigenetic regulation [73], all of which could be examined in future studies. While the present study demonstrates coordinated changes in glycogen content, UDP-glucose levels, NADP^+^/NADPH balance, and key metabolic enzymes, it should be noted that these measurements are based on endpoint analyses and do not directly capture metabolic flux. As such, the precise fate of glycogen-derived substrates, including whether glycogen is preferentially directed toward glycolysis or the pentose phosphate pathway, cannot be definitively determined in the current study. Moreover, although the observed increase in NADPH availability is consistent with enhanced antioxidant capacity, the extent to which pentose phosphate pathway activity contributes causally to this effect remains to be established. These findings support an association between glycogen metabolic remodeling and redox regulation, rather than establishing a fully defined causal pathway. Future studies employing isotope tracing and metabolic flux analysis will be required to delineate the directionality and functional significance of glycogen utilization in this context. In addition, biological variables such as sex and age were not systematically considered in the present study. All experiments were conducted in animals with relatively homogeneous characteristics, which may limit the extent to which the findings capture biological variability. Given that sex- and age-dependent differences can influence ischemic injury severity, metabolic regulation, and functional recovery, future studies should include both sexes and animals of different ages to further strengthen the robustness and translational value of the conclusions.

Another limitation of the present study is the use of undifferentiated SH-SY5Y cells in the OGD/R experiments. SH-SY5Y cells, although derived from a human neuroblastoma lineage, are widely used as an in vitro neuronal model in studies of cerebral ischemia and oxidative stress due to their reproducibility and well-characterized responses to hypoxia and redox injury. Previous studies have successfully employed SH-SY5Y cells to investigate mechanisms of ischemia-induced oxidative damage and ferroptosis-related pathways [74,75]. Nevertheless, SH-SY5Y cells are neuroblastoma-derived rather than primary neurons, and their undifferentiated state may not fully recapitulate the physiological characteristics of mature neurons [76]. In addition, compared with primary neuronal cultures, immortalized cell lines may exhibit altered metabolic properties and stress responses, which could influence their sensitivity to oxygen–glucose deprivation and oxidative injury. Therefore, the in vitro findings should be interpreted with caution. Moreover, SH-SY5Y cells originate from a single donor source, which limits biological heterogeneity and may restrict the generalizability of the results [77]. Future studies employing primary neurons or human iPSC-derived neuronal models will be necessary to further validate and extend these findings. It should be noted that SH-SY5Y cells, particularly in their undifferentiated state, do not fully recapitulate the phenotype of mature neurons. Therefore, the present findings should be interpreted as reflecting neuronal-like cellular responses rather than definitive mechanisms in SH-SY5Y cells. Future studies using primary neurons or human iPSC-derived neurons will be necessary to further validate these observations.

Collectively, our data support a unified mechanistic framework in which EE is associated with SIRT1 to coordinate glycogen metabolic reprogramming, antioxidant defense, ferroptosis suppression, and mitochondrial quality control, ultimately culminating in sustained neuronal survival and functional recovery after CIRI. Rather than acting through isolated pathways, EE appears to promote metabolic and redox homeostasis at a systems level, positioning SIRT1 as a central metabolic hub that integrates energy balance with resistance to lipid peroxidation-driven cell death. Notably, this study reveals an unrecognized link between glycogen metabolism and ferroptosis in the ischemic brain, thereby expanding the conceptual landscape of EE-mediated cerebroprotection beyond synaptic plasticity and neurogenesis. By identifying SIRT1 as a key upstream regulator of this axis, our findings provide mechanistic rationale for targeting metabolic resilience as a therapeutic strategy in acute stroke. From a translational perspective, these results suggest that interventions capable of mimicking or potentiating EE-induced metabolic remodeling—such as SIRT1 activators, NAD^+^-boosting agents, or structured behavioral paradigms—may represent feasible adjunctive approaches to current reperfusion therapies. Given that metabolic instability and ferroptosis are shared pathological features across multiple neurological disorders, the EE–SIRT1–glycogen metabolism–ferroptosis axis identified here may have broader relevance beyond ischemic stroke, offering a conceptual foundation for developing metabolism-centered neuroprotective strategies [78].

## 5. Conclusions

In summary, this study shows that an enriched environment (EE) mitigates ferroptosis-related changes and is associated with improved neurological function following cerebral ischemia–reperfusion injury. These effects are associated with SIRT1 activation and involve the AKT/GSK3β and Nrf2/HO-1 signaling pathways, along with changes in glycogen metabolism, oxidative stress, and ferroptosis-related processes. Importantly, pharmacological inhibition of SIRT1 attenuated the protective effects of EE, supporting its involvement in these responses. Collectively, these findings suggest that EE may represent a potentially relevant non-pharmacological approach in the context of CIRI and highlight metabolic remodeling and ferroptosis-related processes as interconnected areas for further investigation in post-stroke recovery.

## Data Availability

The original contributions presented in this study are included in the article/Supplementary Materials. Further inquiries can be directed to the corresponding authors.

## Supplementary Materials

The following supporting information can be downloaded at: https://www.mdpi.com/article/10.3390/antiox15050570/s1, Table S1: Primary antibodies used in the research.
