# Supplementary material for: Enriched Environment Suppresses Neuronal Ferroptosis Through SIRT1/AKT/GSK3β-Dependent Glycogen Metabolic Reprogramming After Cerebral Ischemia–Reperfusion
Source: Antioxidants (Basel). 2026 Apr 30;15(5):570. doi: 10.3390/antiox15050570 (PMC13203480; doi:10.3390/antiox15050570)
Supplement: Supplementary file 1 [file antioxidants-15-00570-s001.zip › antioxidants-4242505-supplementary.pdf]

Supplementary materials

Table S1. Primary antibodies used in the research

Antibody	Source	Catalog No.	Type	Dilution
ACSL4	CST	38493	Rabbit mAb	1:1000(W.B.)
GPX4	Proteintech	67763-1-lg	Rabbit mAb	1:1000(W.B.)<br>1:200(IF)
GSK3β	CST	9315	Rabbit mAb	1:1000(W.B.)
p-GSK3β	CST	5558	Rabbit mAb	1:1000(W.B.)<br>1:200(IF)
PYGB	Proteintech	12075-1-AP	Rabbit pAb	1:1000(W.B.)
GYS1(phospho S641)	Abcam	Ab314028	Rabbit mAb	1:1000(W.B.)<br>1:200(IF)
SIRT1	CST	9475	Rabbit mAb	1:1000(W.B.)
AKT	CST	9272	Rabbit mAb	1:1000(W.B.)
Phospho-Akt (Ser473)	CST	4060	Rabbit mAb	1:1000(W.B.)
Nrf2	Abcam	ab62352	Rabbit mAb	1:2000(W.B.)<br>1:200(IF)
HO-1	Abcam	ab68477	Rabbit mAb	1:1000(W.B.)<br>1:200(IF)
DHODH	Abcam	ab174288	Rabbit mAb	1:1000(W.B.)
FSP1	CST	51676	Rabbit mAb	1:1000(W.B.)
Histone H3	Abcam	ab1791	Rabbit mAb	1:1000(W.B.)
β-Actin	Abcam	ab8226	Mouse mAb	1:5000(W.B.)
GAPDH	Abcam	ab8245	Mouse mAb	1:5000(W.B.)
α-Tubulin	Abcam	ab7291	Mouse mAb	1:5000(W.B.)
